# Newborn screening for SCID and severe T- and B-cell lymphopenia in Ukraine: the first analysis of the results, 2022–2025

**DOI:** 10.3389/fimmu.2025.1709657

**Published:** 2025-12-11

**Authors:** Oksana Boyarchuk, Halyna Makukh, Alla Volokha, Anastasiia Bondarenko, Nataliia Mytsyk, Oksana Barvinska, Ivanna Shymanska, Yuliia Pohuliai, Mykola Veropotvelyan, Anastasia Haviley, Marharyta Hurina, Yaryna Romanyshyn, Maryna Trophymova, Iryna Hrabovska, Oksana Malko, Tetiana Tsanko, Oksana Tykholaz, Oleksandr Lysytsia, Nataliia Samonenko, Nataliia Olkhovych

**Affiliations:** 1 Department of Children’s Diseases and Pediatric Surgery, I. Horbachevsky Ternopil National Medical University, Ternopil, Ukraine; 2 Department of Regional Center of Neonatal Screening, Lviv Regional Clinical Perinatal Centre, Lviv, Ukraine; 3 Department of Pediatrics, Neonatology, Infectious Diseases, Immunology and Allergology, Shupyk National Healthcare University of Ukraine, Kyiv, Ukraine; 4 Outpatient and Diagnostic Department, National Specialized Children’s Hospital “OHMATDYT”, Kyiv, Ukraine; 5 Department of Pediatrics, Immunology, Infectious and Rare Diseases, International European University, Kyiv, Ukraine; 6 Department of Dermatology, Allergology, Clinical and Laboratory Immunology, Shupyk National Healthcare University of Ukraine, Kyiv, Ukraine; 7 Center of Genetic Diagnostics and Cell Immunotherapy, National Specialized Children’s Hospital “OHMATDYT”, Kyiv, Ukraine; 8 Department of Neonatal Screening, Multiregional Center of Medical Genetics and Prenatal Diagnostics Named After P. M. Veropotvelyan, Kryvyi Rih, Ukraine; 9 Laboratory of Molecular Diagnostics, Kharkiv Specialized Medical Genetics Center – Center for Rare (Orphan) Diseases, Kharkiv, Ukraine; 10 Clinic of Pediatric Immunology and Rheumatology, Western Ukrainian Specialized Children’s Medical Centre, Lviv, Ukraine; 11 Center of Orphan Diseases and Gene Therapy, National Specialized Children’s Hospital “OHMATDYT”, Kyiv, Ukraine; 12 Department of Pediatrics Oncohematology, Volyn Regional Territorial Mother and Child Health Care Center, Lutsk, Ukraine; 13 Center for Oncohematology, Immunology and Metabolic Disorders, Rivne Regional Council Municipal Enterprise “Rivne Regional Children’s Hospital”, Rivne, Ukraine; 14 Department of Oncohematology, Municipal Non-Profit Enterprise “Regional Children’s Hospital of the Transcarpathian Regional Council”, Uzhhorod, Ukraine; 15 Department of Propedeutics of Pediatric Diseases with Patient Care, National Pirogov Memorial Medical University, Vinnytsya, Ukraine; 16 Department of Bone Marrow Transplantation and Intensive Megadose Chemotherapy and Immunotherapy, National Specialized Children’s Hospital “OHMATDYT”, Kyiv, Ukraine

**Keywords:** newborn screening, TREC, KREC, severe combined immunodeficiency, inborn errors of immunity, NBS, SCID, Ukraine

## Abstract

**Introduction:**

Severe combined immunodeficiency (SCID) and other profound T- and B-cell lymphopenias are life-threatening conditions that benefit from early diagnosis and treatment. In October 2022, Ukraine launched a nationwide newborn screening (NBS) program for SCID using the T-cell receptor excision circle/kappa-deleting recombination excision circle/spinal muscular atrophy (TREC/KREC/SMA) assay, despite ongoing war-related challenges. The aim of this study was to analyze the results of the SCID NBS program in Ukraine, evaluate its effectiveness, and outline the current challenges and future directions for its development.

**Methods:**

We analyzed data of screened newborns for SCID and related lymphopenias using the TREC/KREC/SMA assay from October 2022 to April 2025. The results of lymphocyte flow cytometry values, genetic testing, and clinical management of patients with positive TREC/KREC results were evaluated.

**Results:**

Among 398,415 screened newborns, 57 were identified with positive results (32 TREC ± KREC and 25 only KREC). The program demonstrated a high diagnostic yield, with an overall referral rate of 0.01%. In total, 18 newborns with inborn errors of immunity were diagnosed due to NBS (7 SCID/leaky SCID and 11 non-SCID). One case of ZAP70 deficiency was missed due to normal levels of T cells. The incidence of SCID/leaky SCID detected by NBS was 1 in 57,000 live births, and 1 in 49,800 live births when all diagnosed cases, including one initially missed case, were taken into account, which is comparable to data from other countries. All patients with SCID/leaky SCID identified by NBS received hematopoietic stem cell transplantation, with a survival rate of 85.7%. Nijmegen breakage syndrome was the most common syndromic cause of non-SCID T-cell lymphopenias (three cases). The use of the KREC assay enabled the first-time identification in Ukraine of B-cell lymphopenias associated with variants in *IGLL1* gene.

**Conclusions:**

The nationwide NBS program in Ukraine demonstrated high sensitivity and specificity in detecting SCID, with a low referral rate and high survival rates among diagnosed patients.

## Introduction

Severe combined immunodeficiency (SCID) is one of the most critical forms of inborn errors of immunity (IEIs). Without early diagnosis and adequate treatment, including hematopoietic stem cell transplantation (HSCT) or gene therapy, SCID typically leads to death within the first few months of life (1, 2). The disease is characterized by profound lymphopenia and functional impairment of T lymphocytes, resulting in an extremely high risk of severe infections shortly after birth. Early diagnosis enables pre-symptomatic or early-age HSCT, which significantly improves survival and long-term outcomes in affected children (3).

One of the most effective strategies for early SCID detection is newborn screening (NBS) based on the quantification of T-cell receptor excision circles (TRECs) from dried blood spots (DBSs) (4). Additionally, the measurement of kappa-deleting recombination excision circles (KRECs) enables the identification of infants with severe B-cell lymphopenia (5). To date, SCID screening has been recommended and implemented in many countries worldwide, including the United States, some European countries, Japan, and Israel, demonstrating its effectiveness in detecting not only SCID but also other types of significant T- and B-cell lymphopenias (6, 7).

In Ukraine, a national NBS program for SCID and related lymphopenic disorders was officially launched in 2022 as part of the broader expansion of neonatal screening. This initiative marked a major step toward improving early diagnosis and clinical care for affected children (8). A preceding pilot project had already demonstrated the feasibility and diagnostic value of this approach, successfully identifying not only SCID but also other lymphopenic conditions such as Nijmegen breakage syndrome and ataxia-telangiectasia (9–11).

The aim of this study was to analyze the initial results of the SCID NBS program in Ukraine, evaluate its effectiveness, and outline the current challenges and future directions for its development.

## Materials and methods

### Study design

Expanded NBS for 21 disorders, including SCID and severe T- and B-cell lymphopenias using the TREC/KREC assay, was initiated in Ukraine in October 2022 at two regional centers (Kyiv and Lviv). In April 2023, the program was scaled up to the national level with the addition of two further centers (Kharkiv and Kryvyi Rih), thereby covering the entire territory of Ukraine except for areas temporarily occupied by Russia. Each center is responsible for screening in several surrounding regions, with distribution determined by geographical location, logistical feasibility, and laboratory capacity.

This study includes data from all newborns screened since the launch of the program in October 2022 up to 1 April 2025, based on results collected from the four Ukrainian NBS centers.

### Participants

Screening was performed in newborns who had reached a gestational age of at least 32 weeks, within 48–72 h of life. Preterm infants born at <32 weeks were screened after reaching 36 weeks of gestational age or during preparation for discharge from the neonatal unit (2–3 days before the planned discharge). This approach follows national recommendations and aims to minimize false-positive results related to transient lymphopenia frequently observed in very preterm infants during the early postnatal period. In addition, validated TREC/KREC cutoff values for extremely preterm neonates are currently lacking, which further supports delaying testing until immune parameters stabilize. Blood collection was routinely carried out at 48–72 h of life; in cases of severe clinical condition (e.g., shock and hemorrhage), sampling was postponed until after stabilization.

Following confirmation of eligibility criteria, written informed consent was obtained from one parent or a legal guardian. DBS samples were collected on Whatman™ 903 filter paper cards and sent to the designated regional neonatal screening center within 24 h of collection.

### Screening workflow

First-line screening was performed using quantitative real-time polymerase chain reaction (PCR) with Biocore TREC/KREC/SMA (spinal muscular atrophy) kits (Biocore Technology, Ukraine) to measure TREC and KREC levels in DBS, serving as markers of T- and B-lymphocyte counts, respectively.

DNA was extracted and purified from DBS collected on filter paper using the Biocore^®^ Nucleo-M Plus kit. Quantification of TREC, KREC, and SMN1 was carried out with the Biocore^®^ SMA/SCID assay on a QuantStudio Real-Time PCR (qPCR) system. This workflow allows simultaneous detection of TREC, KREC, SMN1, and the internal control gene RNase P within a single reaction tube.

Cutoff values were defined according to the manufacturer’s instructions, based on the number of TREC or KREC copies per 10^6^ cells. Each regional neonatal screening center determined the cutoff values based on its own dataset. The average cutoff value for TREC was 5,395 copies per 10^6^ cells (99.5 percentile); for KREC, 1,312 copies per 10^6^ cells (99.9 percentile).

Positive first-line results were referred to a clinical geneticist (regional NBS coordinator), who informed the parents by phone and organized confirmatory testing.

### Diagnostic verification

**Figure 1:**
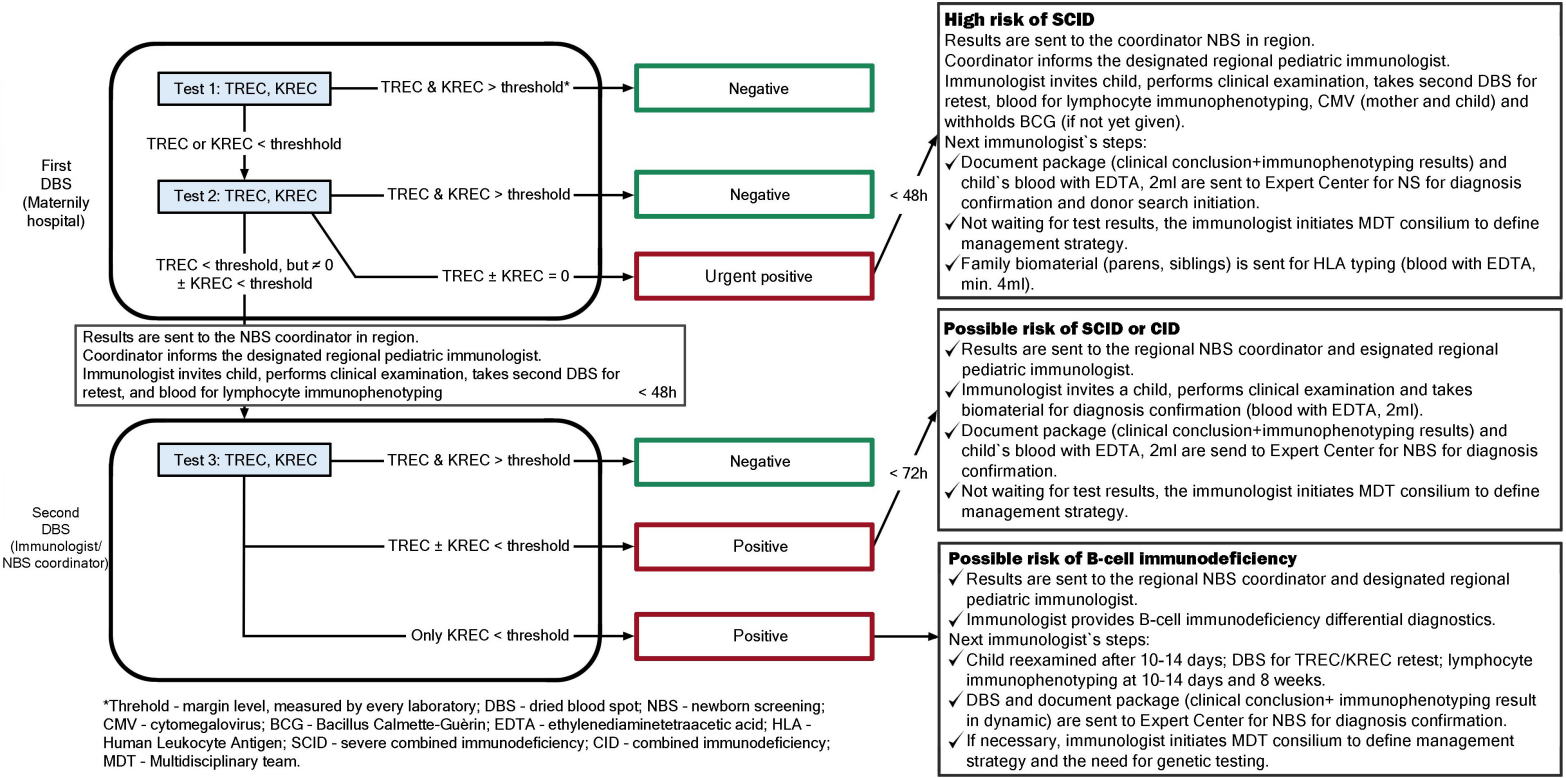
Patient pathway following a positive screening result.

An expert group developed a clinical pathway for infants with positive NBS results for SCID and other severe T- and B-cell lymphopenias, building on the recommendations of Swiss experts (12) and the findings of the preceding Ukrainian pilot project (10) (**Figure 1**).

In cases of an urgent positive screening result from the initial DBS (TREC ± KREC = 0), the risk of SCID is considered high. Within 48 h, a notification is sent to the regional NBS coordinator, who informs the designated regional pediatric immunologist. The immunologist contacts the family, evaluates the infant’s clinical condition, and arranges further testing: a repeat DBS sample for retesting, urgent lymphocyte immunophenotyping, and cytomegalovirus (CMV) testing of both mother and infant (12). The immunologist also determines whether BCG vaccination should be contraindicated if it has not yet been administered.

Subsequently, the immunologist forwards the documentation, test results, and the infant’s biological material (EDTA blood) to the National Expert Center for Neonatal Screening for genetic confirmation of the diagnosis and initiation of donor search. At the same time, biological samples from family members (parents and siblings) are submitted for human leukocyte antigen (HLA) typing. The immunologist convenes a multidisciplinary team meeting to determine the treatment strategy for the patient.

A similar algorithm applies when the repeat DBS sample is positive (TREC ± KREC below the cutoff). Venous blood samples are collected in EDTA tubes for flow cytometry analysis to determine T-, B-, and NK-cell counts, performed either in regional laboratories or at the national reference laboratory of “OHMATDYT” Children’s Hospital, Ministry of Health of Ukraine, for diagnostic verification.

Only when KREC values fall below the cutoff is suspicion of a B-cell immunodeficiency raised. The regional NBS coordinator informs the responsible immunologist, who initiates differential diagnosis to exclude possible secondary causes. The child undergoes clinical examination and serial lymphocyte immunophenotyping. In cases of persistent B-cell lymphopenia, the immunologist initiates a multidisciplinary consultation and molecular genetic testing.

Genetic diagnostics are performed at the Laboratory of Medical Genetics, National Children’s Specialized Hospital “OHMATDYT”, Ministry of Health of Ukraine (National Expert Center for Neonatal Screening), or at other certified genetic laboratories if urgent testing is not required. Genetic testing of rearrangements in genes implicated in SCID and other severe T- and B-cell lymphopenias is conducted using next-generation sequencing (NGS) with custom panels [72 genes associated with SCID and combined immunodeficiencies (CID), and 404 genes associated with IEI] or whole-exome sequencing (WES).

Patients are monitored with follow-up visits every 3 months during the first year of life and every 6 months during the second year. All data are entered into the electronic registry of neonatal SCID screening.

### Statistical analysis

This study was designed as a population-based prospective screening study. Time to treatment initiation was defined as the interval between the date of birth and the start of therapy. Statistical analyses were performed using STATISTICA 10. Categorical variables are presented as absolute numbers and percentages, and comparisons were made using the chi-squared test. Continuous variables were assessed for normality using the Kolmogorov–Smirnov or Shapiro–Wilk tests. Data are expressed as median and interquartile range (IQR), as appropriate. Differences in continuous variables between groups were evaluated using the Kruskal–Wallis test. A two-sided *p*-value of <0.05 was considered statistically significant.

## Results

### Baseline characteristics of newborns with positive screening results

From 17 October 2022 to 1 April 2025, a total of 398,415 newborns were screened. Among the regional centers, the highest number of screened neonates was recorded in the Lviv and Kyiv centers, which started operating 6 months earlier and cover the western and central regions of Ukraine, respectively (**Table 1**).

**Table 1:** Number of screened neonates and SCID incidence in regional centers.

Indicator	Lviv	Kyiv	Kryvyi Rih	Kharkiv	Total
Period	17 October 2022 to 01 April 2025	17 October 2022 to 01 April 2025	01 May 2023 to 01 April 2025	24 April 2023 to 01 April 2025	17 October 2022 to 01 April 2025
Number of months	29.5	29.5	23	23.25	29.5
Number of screened	141,730	130,377	70,720	55,588	398,415
New sample test TREC only/TREC+KREC/KREC only	230 (0.16%) (73/28/129)	114 (0.09%) (36/10/68)	44 (0.06%) (2/6/36)	55 (0.10%) (1/5/49)	443 (0.11%) (116/45/282)
Referral to confirm/rule out diagnosis TREC only/TREC+KREC/KREC only	25 (0.02%) (3/10/12)	20 (0.02) (5/8/7)	8 (0.01%) (1/4/4)	4 (0.01%) (0/1/2)	57 (0.01%) (9/23/25)
Rate of the referral	1:5,570	1:6,520	1:13,900	1:13,900	1:7,000
SCID, *n*	4	1	1	1	7
Incidence	1:35,000	1:130,000	1:70,000	1:55,000	1:57,000

SCID, severe combined immunodeficiency.

A total of 443 samples (0.11%) required repeat testing due to low or inconclusive TREC/KREC values, and 57 newborns (0.01%) were referred for confirmatory diagnostics (**Table 1**). The rates of repeat testing and referrals varied slightly across regional centers, ranging from 0.06% to 0.16% and 0.01% to 0.02%, respectively.

In total, 57 infants had a positive screening result. Among them, 32 cases were positive for TREC ± KREC, including 4 with urgent positivity; 7 of these were diagnosed with SCID or leaky SCID. In the remaining 25 infants, low TREC and/or KREC levels were attributable to other non-SCID conditions, including syndromic T-cell lymphopenia (TCL), idiopathic transient TCL, secondary TCL, and prematurity. An additional 25 infants had positive results for KREC only. Baseline characteristics of neonates with positive NBS results are summarized in **Table 2**.

**Table 2:** Baseline characteristics and follow-up testing (immunologic and genetic) of neonates with positive NBS for TREC/KREC.

Characteristic	Positive TREC ± KREC			Positive KREC	Total
	SCID/leaky SCID	Non-SCID TCL and other			
	*n* = 7	*n* = 25		*n* = 25	*n* = 57
Male/Female	6/1 (85.7/14.3)	16/9 (64/36)		16/9 (64/36)	38/19 (66.7/33.3)
Gestational age					
Extreme prematurity (less than 28 weeks)	0 (0)	6 (24)		1 (4)	7 (12.3)
Very preterm (28–32 weeks)	0 (0)	7 (28)		0 (0)	7 (12.3)
Moderate preterm (32–37 weeks)	1 (14.3)	6 (24)		8 (32)	15 (26.3)
At term (GA ≥ 38 weeks)	6 (85.7)	6 (24)		16 (64)	28 (49.1)
Birth weight					
Less than 1,000 g	0 (0)	4 (16)		0 (0)	4 (7)
1,000–1,499 g	0 (0)	9 (36)		1 (4)	10 (17.5)
1,500–2,499 g	0 (0)	5 (20)		4 (16)	9 (15.8)
≥2,500 g	7 (100)	7 (28)		20 (80)	34 (59.6)
Immunologic testing	7 (100)	19 (76)		24 (96)	50 (87.7)
Genetic testing	7/7 (100)	13/15 (86.7)		13/13 (100.0)	33/35 (94.3)

NBS, newborn screening; GA, gestational age; SCID, severe combined immunodeficiency; TCL, T-cell lymphopenia.

Results are presented in *n* (%); % of genetic testing is shown for newborns with confirmed T-cell lymphopenia (CD3 ≤ 1,500 cells/µL) or suspected SCID/CID, B-cell lymphopenia (CD19 ≤ 100 cells/µL).

It is noteworthy that among newborns with positive screening results, male infants predominated at a ratio of 2:1 (*p* = 0.0004). The difference was most pronounced in the cohort of infants diagnosed with SCID, where the male-to-female ratio reached 6:1. Among all screen-positive infants, 50.9% were preterm, whereas in the SCID group, only one infant (14.3%) was moderately preterm.

The highest proportion of preterm newborns was observed among the group with positive TREC ± KREC results (non-SCID TCL and other), where 76% of newborns were preterm. This group included six extremely preterm, seven very preterm, and six moderate preterm infants. In contrast, among newborns with positive KREC results only, term infants predominated (64%). Regarding the timing of screening, among TREC ± KREC-positive newborns, six moderately preterm infants were screened within the first days after birth according to protocol. In 10 newborns, the first DBS sample was collected at 33–36 weeks corrected gestational age (GA): 2 at 36 weeks and 8 at 33–35 weeks, prior to discharge, with a second DBS collected at 36–38 weeks corrected GA. Three newborns were screened earlier than recommended (28–31 weeks GA) due to urgent clinical indications or imminent transfer, and repeat samples were collected later.

A birth weight of ≥2,500 g was recorded in the majority of infants (59.6%), and in all newborns diagnosed with SCID/leaky SCID (100%). Low birth weight was most frequently observed in the non-SCID TCL and other TREC ± KREC-positive groups: four infants weighed <1,000 g, nine infants weighed 1,000–1,499 g, and five infants weighed 1,500–2,499 g.

Immunologic evaluation was performed in 50/57 (87.7%) infants with a positive TREC/KREC screening result. Four infants died before confirmatory testing could be completed, one infant from a frontline region was lost to follow-up, and the parents of two infants declined further testing. The TREC/KREC values in DBS and immunophenotyping results in newborns with positive NBS are presented in **Supplementary Table S1 (Supplementary Materials)**.

Genetic testing was carried out in 33/57 (57.9%) of all screen-positive infants and in 33/35 (94.3%) of those with confirmed T- and/or B-cell lymphopenia on immunophenotyping or with clinical suspicion of IEI. Pathogenic variants associated with T- and B-cell lymphopenia were identified in 18/33 (54.5%) of genetically tested newborns with positive screening results.

### SCID incidence and spectrum

In total, seven cases of SCID were identified (five typical and two leaky), corresponding to an overall incidence of 1:57,000. The highest incidence was observed in the western region (1:35,000), with three children (42.9%) originating from the Volyn Polissia area (**Table 1**). All infants with suspected SCID underwent genetic testing.

The spectrum of identified SCID cases is summarized in **Table 3**. The most frequent diagnosis was typical SCID associated with variants in the *IL2RG* gene (three cases). Additional cases were associated with variants in *IL7R* and *AK2* genes (one case each). Leaky SCID was linked to variants in the *RAG1* and *PNP* genes.

**Table 3:** Characteristic of infants with confirmed cases of SCID.

No.	Year of birth	GA, week	Weight, g	Gender	TREC result	KREC result	Phenotype	CD3/СD19/NK, cells/µL	Affected gene/variant	Clinical diagnosis	HSCT	Age at HSCT, months	Donor	Outcome
1	2023	39	3,310	M	Positive	Negative	Т-B+NK+	10/1,208/762	***IL2RG*** c.270-1G>A	SCID	Yes	2	Related	Alive, well
2	2024	40	3,315	M	Urgent positive	Negative	Т-B+NK−	0/930/90	***IL2RG*** c.578G>A	SCID	Yes	4.5	Unrelated	Alive, well
3	2024	40	3,200	M	Positive	Negative	Т-B+NK−	18/223/41	***IL2RG*** c.836G>A	SCID	Yes	3,5	Unrelated	Alive, well
4	2024	40	3,520	M	Positive	Positive	T-B-NK−	500/80/60	***PNP*** c.751delA	Leaky SCID	Yes	6	Unrelated	Alive, well
5	2024	40	3,390	M	Positive	Positive	T-B-NK+	120/60/1,460	***RAG1*** c.256_257delAA	Leaky SCID	Yes	5	Unrelated	Alive, well
6	2025	40	3,900	F	Urgent positive	Positive	T-B+NK+	0/1,100/800	***IL7R*** c.132C>A	SCID	Yes	2.5	Related	Alive, well
7	2025	37	2,630	M	Positive	Positive	T-B+NK−	60/310/10	***AK2*** c.84dup	SCID	Yes	3.5	Unrelated	Deceased on the 12th day after HSCT

GA, gestational age; M, male; F, female; SCID, severe combined immunodeficiency; HSCT, hematopoietic stem cell transplantation.

All infants diagnosed with SCID underwent HSCT between the ages of 2 and 4.5 months for typical SCID and by 6 months for leaky SCID (**Table 2**). Notably, except for one case of leaky SCID associated with a *PNP* gene variant, all HSCTs were performed in Ukraine at the Bone Marrow Transplantation Department of the National Children’s Specialized Hospital “OHMATDYT”. Two HSCTs were performed from related donors (at ages 2 months and 2 months 3 weeks), while the remaining transplants were performed from unrelated donors. All HSCTs resulted in successful engraftment and immune reconstitution, except for one child with reticular dysgenesis associated with an *AK2* gene variant, who underwent unrelated donor HSCT at 3 months 2 weeks but died on day 12 post-transplant due to infectious complications. The overall survival following HSCT was 85.7%.

No SCID cases were missed, except for ZAP70 deficiency, a form characterized by normal T-cell counts but impaired T-cell function. Immunodeficiency was suspected in this patient due to an adverse reaction to BCG vaccination (left axillary lymphadenitis), generalized lymphadenopathy involving mediastinal and mesenteric nodes, dermatitis, and anemia. Immunophenotyping at 8 months showed normal counts of CD3, CD4, and CD19 lymphocytes (5,830/µL, 4,640/µL, and 2,710/µL, respectively) with a slightly reduced percentage of CD8 cells (13%), though their absolute number was within the normal range (1,250/µL). Genetic testing revealed the c.392G>A (p.Trp131*) variant in the *ZAP70* gene. The child subsequently developed disseminated BCG infection with involvement of axillary, mesenteric, and mediastinal lymph nodes. She underwent successful allogeneic HSCT in Germany at the age of 1 year 2 months. Mild graft-versus-host disease was observed during the first post-transplant month. The child remains on antimycobacterial therapy for BCG infection.

### Non-SCID low TREC ± KREC

In addition to the seven confirmed SCID cases, 25 newborns with low TREC ± KREC levels were identified (**Supplementary Table S2, Supplementary Materials**). Among them, one infant carried a heterozygous c.1233delG (p.Leu413TrpfsTer137) variant in *FOXN1* gene. Flow cytometry revealed the following lymphocyte subsets: CD3—190 cells/µL, CD4—170 cells/µL, CD4/CD45RA^+^/CD45RO^−^—10%, CD4/CD45RA^–^/CD45RO^+^—47%, CD8—5 cells/µL, and CD19—820 cells/µL. Following expert consultation, a management strategy of close observation was adopted. The child has been on immunoglobulin replacement therapy since the age of 5 months. Up to 1 year of age, the patient received antimicrobial prophylaxis with co-trimoxazole and isoniazid. At present (1 year 6 months), the patient is clinically stable with mild undernutrition, a history of mild upper respiratory tract infections, and four episodes of acute gastroenteritis.

Three infants were diagnosed with Nijmegen breakage syndrome, all of whom demonstrated persistently low TREC/KREC levels across two DBS samples, accompanied by decreased CD3, CD4, and CD19 counts (**Supplementary Table S2, Supplementary Materials**).

Three newborns with syndromic features died before confirmatory flow cytometry could be performed. In two of them, the clinical presentation was consistent with 22q11.2 deletion syndrome (DS); however, the limited amount of DBS material did not allow fluorescence *in situ* hybridization (FISH) analysis or multiplex ligation-dependent probe amplification (MLPA) to be conducted. NGS with custom panels performed in one of these cases did not reveal any pathogenic variants.

In total, eight infants died between the ages of 1 and 7 months, the majority (75%) before 3 months of age. Of these, seven had positive TREC/KREC results, and one had an isolated positive TREC result. Among these, six were preterm infants: two extremely preterm (born at 24–25 weeks of gestation, weighing 570 and 750 g, respectively) and three very preterm (28–32 weeks). One term infant was small for GA (birth weight, 2,200 g). Reported causes of death included severe congenital heart defects (three cases), severe infections, including intrauterine infections (four cases), and neurological complications (one case). In most infants, multiple comorbidities (e.g., intrauterine infection, neonatal sepsis, and neurological complications) coexisted against the background of prematurity.

Secondary TCL was attributed to congenital heart disease (CHD) in three infants and hydrops in one case. Prematurity as the primary cause of low TREC levels was observed in 11 infants (44%), though only 3 of them had confirmed TCL with CD3 ≤ 1,500 cells/µL.

Overall, three infants were lost to follow-up: one from a frontline region and two who relocated abroad. In one case, the parents declined further investigations due to the satisfactory clinical condition of the child. One case (4%) was ultimately confirmed to be a false-positive result.

Across this group, 11 newborns had CD3 counts below 1,500 cells/µL. Including the 7 infants with confirmed SCID/leaky SCID, a total of 18 infants met the criteria for TCL, corresponding to an incidence of 1:22,000. Immunologic testing could not be performed in six infants due to early death before confirmatory evaluation (*n* = 4), parental refusal (*n* = 1), or loss to follow-up (*n* = 1). In one case, the immunogram was performed, but the results could not be retrieved as the child died soon after testing, the treating physician relocated, and contact with the family was lost.

Genetic testing was performed in 13 patients, with clinically significant variants identified in 4 children: 1 with *FOXN1* and 3 with *NBN* gene variants. In one infant with multiple brain malformations, growth and developmental delay, congenital hypothyroidism, and combined T- and B-cell lymphopenia (CD3—1,280 cells/µL, CD19—76 cells/µL), a heterozygous variant of uncertain significance (VUS) с.5878Т>C (p.Ser1960Pro) in *CHD7* gene was detected, consistent with a possible diagnosis of CHARGE syndrome. Two infants were heterozygous carriers of pathogenic variants: one in the *FANCI* gene (c.1622del), associated with autosomal recessive Fanconi anemia type 1, and the other in the *ADA* gene (c.956_960del), associated with SCID. Another child had heterozygous VUS с.5628G>T (p.Glu1876Asp) in the *DOCK8* gene.

### Low KREC, normal TREC

Absent or low KRECs with normal TREC levels were observed in 25 patients, of whom 13 newborns (52%) had completely absent KRECs. Among the three children with absent KRECs, X-linked agammaglobulinemia (XLA) associated with variants in the *BTK* gene was diagnosed (**Supplementary Table S3, Supplementary Materials**). Additionally, four children with absent KRECs and normal TREC levels were found to carry variants in the *IGLL1* gene that is associated with autosomal recessive IGLL1 agammaglobulinemia. All these children had B-cell counts below 100 cells/µL. Two children carried previously reported variants (c.425C>T and c.258del), while the other two were compound heterozygous for one known variant (c.425C>T) and a second novel variant (c.377T>C and c.64C>T).

In four children with low KRECs, transient B-cell lymphopenia was diagnosed. B-cell counts normalized by 12 months of age, and immunoglobulin levels remained within the normal range.

In one child with low KRECs at birth and absent B cells at 1 month, the cause was maternal rituximab therapy during pregnancy. The child received immunoglobulin replacement therapy from 3 months of age for 7 months, and immunophenotyping normalized by 7 months (CD19—20.5%, 1,515 cells/µL).

Prematurity was the main cause of low KRECs in two children. Overall, nine children in this cohort were premature, with only one classified as very preterm.

In four children, the cause of low KRECs could not be determined; B-cell counts were either within the normal range or mildly reduced. These cases were considered false positives (16%). The mother of one child declined immunological evaluation, considering the child healthy.

Genetic testing was performed in 13 children with confirmed B-cell lymphopenia and/or agammaglobulinemia based on immunophenotyping results. Among those tested, three cases of variants in the *BTK* gene and four cases in the *IGLL1* gene were confirmed and one result is pending. Single cases of variants were identified in the *CHEK2, PARN*, and *CEBPE* genes.

A heterozygous VUS c.1514G>A (p.Arg505Gln) was detected in the *PARN* gene, which is associated with both autosomal dominant and autosomal recessive forms of dyskeratosis congenita (DC) spectrum disorders. This patient presented with low B-cell levels (263 cells/µL) and low IgA and IgM levels (0.02 and <0.2 g/L, respectively) at 3 weeks of age, which normalized by 1 year. The *CHEK2* variant is associated with an increased risk of cancer. In another child, a heterozygous VUS c.782G>A (p.Arg261His) in the *CEBPE* gene, associated with specific granule deficiency, was identified. This finding was considered incidental and not related to B-cell lymphopenia, but the child requires ongoing monitoring.

In two cases, no significant variants were detected—one using an IEI gene panel and the other via WES.

### Diagnostic outcomes and immunophenotyping in infants with positive newborn screening

The summarized results of diagnosed conditions in patients with a positive screening are presented in **Table 4**. In total, 18 cases of IEI were diagnosed through NBS, accounting for 31.5% of infants with a positive screening result. The genetic testing result for one additional child is still pending.

**Table 4:** The summarized results of diagnosed conditions in patients with a positive TREC/KREC newborn screening.

Condition	Number of patients
Low/absent TREC or TREC/KREC	32
SCID	5
Leaky SCID	2
Nijmegen breakage syndrome	3
T-cell lymphopenia, infantile (FOXN1)	1
T-cell lymphopenia idiopathic	2
Prematurity	11 (20)*
Congenital heart defects	3
Severe infection	4
Hydrops	1
False positive	1
Lost to follow-up	1
Unknown cause (refusal)	1
Additional findings	
*CHD7*, associated with CHARGE syndrome (VUS)	1
*ADA*, carrier	1
*DOCK8*, carrier (VUS)	1
*FANCI*, heterozygous	1
Low KREC, TREC normal	25
X-linked agammaglobulinemia (*BTK*)	3
B-cell lymphopenia (*IGLL1*)	4
Idiopathic B-cell lymphopenia	2
Idiopathic transient B-cell lymphopenia	5
Transient hypogammaglobulinemia	1
Maternal immunosuppressive medication	1
Prematurity	2 (9)*
Perinatal infection	2
False positive	4
Unknown cause (refusal)	1
Additional findings	
*CHEK2*	1
*PARN* (VUS)	1
*CEBPE*, associated with specific granule deficiency (VUS)	1

SCID, severe combined immunodeficiency; VUS, variant of uncertain significance.

*Indicates in how many cases prematurity was the primary cause of a positive screening result, with the total number of preterm infants in this group given in parentheses.

**Figure 2:**
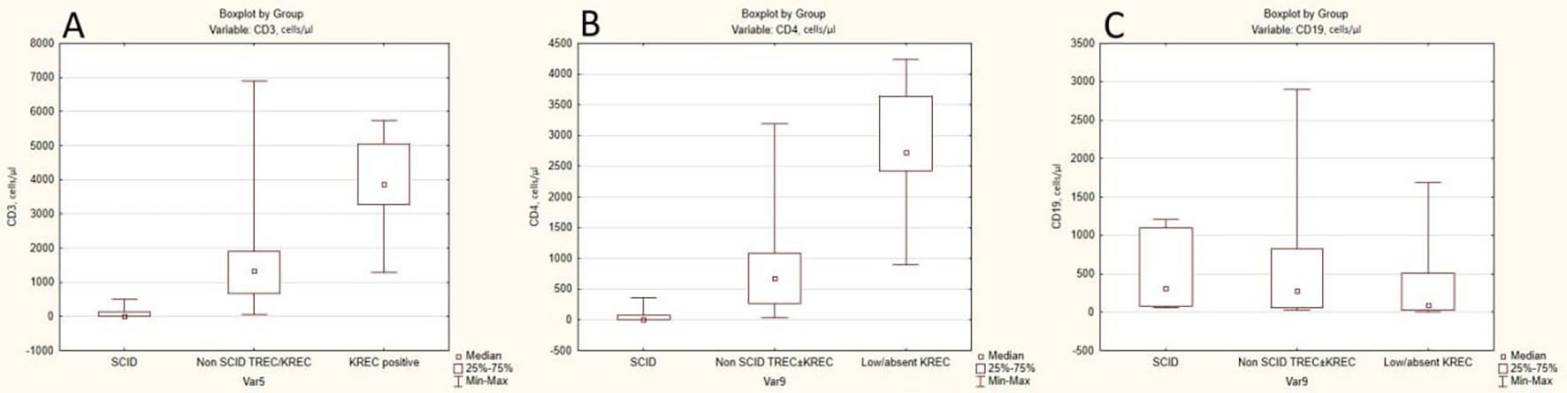
Flow cytometry results for patients with abnormal NBS. The boxplots show the median, interquartile range (25%–75%), and minimum–maximum values. **(A)** Distribution of CD3^+^ T-cell counts in infants with SCID, non-SCID cases with low TREC ± KREC, and KREC-positive cases. **(B)** Distribution of CD4^+^ T-cell counts in infants with SCID, non-SCID cases with low TREC ± KREC, and KREC-positive cases. **(C)** Distribution of CD19^+^ B-cell counts in infants with SCID, non-SCID cases with low TREC ± KREC, and KREC-positive cases.

Lymphocyte immunophenotyping data in infants with abnormal NBS results are presented in **Supplementary Table S1 (Supplementary Materials)**. The median CD3 and CD4 counts in patients with SCID/leaky SCID were 18 [0; 120] cells/µL and 10 [0; 72] cells/µL, respectively. In contrast, in the non-SCID low TREC or TREC/KREC group, the corresponding values were 1,335 [670; 1,907] cells/µL and 670 [267; 1,080] cells/µL. In the group with low/absent KREC, the median CD3 and CD4 counts were 3,877 [3,290; 5,050] cells/µL and 2,730 [2,430; 3,640] cells/µL, respectively. A significant difference between groups was observed for CD3 and CD4 levels (*p* < 0.001), whereas CD19 levels did not differ significantly among groups (310 [80; 1,100] cells/µL; 279 [56; 820] cells/µL; 90 [30; 504] cells/µL, respectively; *p* > 0.05) (**Figure 2**).

## Discussion

The results of the NBS program for SCID and other severe T- and B-cell lymphopenias in Ukraine, implemented from October 2022 to April 2025 and covering 398,415 neonates, demonstrated its effectiveness in identifying SCID/leaky SCID, non-SCID TCL, agammaglobulinemia, and B-cell lymphopenia.

The overall rate of repeat testing (new sample tests) was 0.11%, with center-specific variation ranging from 0.06% to 0.16%, which aligns with findings from other programs (13–15). The referral rate was 0.01% (approximately 1:7,000 screened newborns), again showing minimal variation between screening centers and consistent with other reports (14–17). This low referral rate, coupled with a high diagnostic yield, reflects the sensitivity and specificity of the implemented screening algorithm.

The primary goal of TREC-based NBS is the early detection of SCID. In our cohort, the incidence of SCID detected by NBS was 1 in 57,000 live births, and 1 in 49,800 live births when all diagnosed cases were taken into account, which is comparable to previously published data. For example, during the implementation of SCID screening in California (2010–2017), the reported incidence was 1 in 65,000 newborns (18). In Germany, the incidence of SCID/leaky SCID/Omenn syndrome was 1 in 54,000 (6). Notably, no confirmed SCID cases were missed by the screening, except for a patient with ZAP70 deficiency, which presents with normal T-cell numbers but impaired function and thus may escape detection by TREC-based methods. Consistent with other cohorts (6, 18), *IL2RG* gene variants were the most common genetic cause identified in our patients with SCID.

All children diagnosed with SCID or leaky SCID underwent HSCT. Those with typical SCID received HSCT before 4.5 months of age, and those with leaky SCID received HSCT before 6 months, which aligns with international standards (6, 18). Of the seven transplants performed, six were conducted in Ukraine and one was carried out in Germany. The timing of HSCT was dependent on donor availability. In cases with related donors, transplantation occurred as early as 2 to 2.5 months of age. All HSCTs were successful, except for one case involving a pathogenic variant in the *AK2* gene. This outcome is in line with previously reported data showing a 68% post-HSCT survival rate in patients with *AK2* deficiency, with most deaths occurring within 6 months of transplantation due to infections such as encephalitis, respiratory infections, pulmonary aspergillosis, and systemic adenovirus (19).

In our cohort, the overall survival rate among patients with SCID was 85.7%, which is comparable to outcomes reported in other countries. For instance, survival in patients with SCID identified through NBS programs reached 86% in Switzerland (20), 93% in Israel (21), 94% in California (18), and 96% in Germany (6).

The overall incidence of TCL (defined as CD3^+^ T cells <1,500/μL) was 1 in 22,000, similar to data from Germany (6), but slightly lower than the rate reported in California (1 in 15,300 newborns) (18). In our screened population, Nijmegen breakage syndrome was the most common syndromic cause of TCL (three cases), while in other populations, 22q11.2 DS (DiGeorge syndrome) tends to predominate (6, 17, 18).

The relatively high prevalence of the Slavic founder mutation (c.657_661del5) in the *NBN* gene, particularly in Western regions of Ukraine, likely explains the predominance of Nijmegen breakage syndrome in our cohort and was similarly confirmed in the pilot screening phase (11). Importantly, NBS allowed early identification of Nijmegen breakage syndrome, facilitating early intervention.

The absence of confirmed cases of 22q11.2DS or ataxia-telangiectasia among children with TCL raises some concern and highlights the need for deeper clinical–genetic correlation and long-term follow-up. Notably, among deceased patients, three had congenital heart defects and three had thymic hypoplasia or athymia. In one of these cases, 22q11.2DS was diagnosed postmortem during autopsy, but could not be genetically confirmed, as only panel genetic testing was feasible.

Considering the estimated prevalence of 22q11.2DS (1:3,000–6,000 live births) (22), at least 65 cases would be expected to occur annually in Ukraine. Although not all affected newborns present with TCL detectable by TREC screening, the complete absence of confirmed cases in our cohort suggests potential underdiagnosis. A similar pattern was observed in the Czech Republic, where among 200,000 screened newborns, only seven cases were identified (17). This underrepresentation may be partly related to higher TREC cutoff thresholds, which can miss 22q11.2DS cases with milder lymphopenia. Additionally, the patient carrying a DOCK8 VUS and presenting with athymia might represent a misclassified case, in whom 22q11.2DS could not be excluded.

Among the infants with positive TREC ± KREC screening results but without SCID, the majority (76%) were preterm neonates. However, in only three of these cases was prematurity considered the sole cause of TCL (CD3 < 1,500 cells/µL), suggesting additional underlying factors requiring further evaluation.

The implementation of the KREC assay significantly enhanced the diagnostic capacity for B-cell-related immunodeficiencies. It enabled the early detection of three cases of XLA and four cases of B-cell lymphopenia associated with *IGLL1* variants. These *IGLL1*-related disorders had not previously been identified in Ukraine, highlighting the added diagnostic value of KREC testing (23). Evidence from international patient surveys supports the importance of early XLA diagnosis, showing that patients with an early diagnosis experience fewer severe infections and reduced hospitalization, and report a quality of life comparable to the general population (24).

The false-positive rate of KREC results was 16%, which is lower than that reported in other studies (55%) (17). This difference may reflect population-specific factors, such as higher maternal medication rates in the referenced cohort, rather than methodological differences.

### Challenges and perspectives

Analysis of the 2.5-year nationwide NBS program for SCID and other severe T- and B-cell lymphopenias in Ukraine not only confirms its effectiveness in early diagnosis and improved survival of children with SCID and other IEIs, but also highlights several ongoing challenges and opportunities for further improvement.

One of the central issues relates to TREC and KREC quantification and the determination of threshold (cutoff) values for NBS for SCID and other severe T- and B-lymphopenias. The absence of universally accepted cutoffs complicates interpretation, as laboratories often rely on manufacturer-specific thresholds. Experience with the BIOCORE^®^ SMA/SCID commercial test system revealed several limitations that are likely representative of broader challenges in the field. The current reliance on cycle threshold (Ct) values exceeding 37 as indicators of positive results, while ensuring high sensitivity, may compromise specificity and increase false-positive rates, particularly in samples with suboptimal DNA quality or quantity.

Moreover, the interpretation of absent versus decreased TREC/KREC levels requires different analytical approaches. While the absence of these markers provides relatively clear interpretive guidance, decreased levels present significant challenges that cannot be adequately addressed without proper normalization and quality assessment protocols. The absolute quantification approach, using diluted plasmid standards spanning 30 to 3,000,000 copies, provides an adequate dynamic range for most clinical scenarios. However, interpretation becomes substantially more complex in borderline cases, where multiple confounding factors, such as sample quality, PCR efficiency, and pre-analytical variables, may significantly influence results. This challenge is particularly pronounced given that genetic confirmation was not achievable in all cases of T-cell and B-cell lymphopenia.

To enhance SCID and other IEI screening, incorporating standards with known plasmid copy numbers for RNaseP, TREC, and KREC sequences could provide a comprehensive and highly effective approach (25).

One of the most significant limitations identified in current protocols is the lack of quantitative assessment of the RNaseP (RNP) gene, which serves as an internal control for sample adequacy and DNA input. While RNP amplification is included in the reaction mixture, its quantification is not standardized, representing a missed opportunity for result normalization. The absence of this normalization step creates substantial interpretive challenges, particularly when distinguishing between true immunodeficiency and technical artifacts related to sample quality or DNA degradation.

Our analysis suggests that implementing RNP quantification and expressing TREC/KREC results as copy numbers per 1,000,000 cells would significantly improve assay reliability and clinical interpretation. Based on our findings, we propose improvements to current TREC/KREC quantification protocols. First, the mandatory inclusion of quantitative RNP assessment, with standardized calculation of TREC/KREC copy numbers per 1,000,000 cells, should be implemented across these testing platforms. Next, comprehensive validation studies should be conducted to establish reference ranges that account for the physiological variations in lymphocyte development during the neonatal period.

Genetic confirmation was not possible in all cases of TCL and BCL, primarily due to war-related disruptions, including patient displacement, damaged infrastructure, and limited laboratory capacity (26). Four children (two with TCL and two with BCL) moved abroad, and follow-up was lost. One child from a frontline region could not be properly assessed due to logistical breakdowns. Some children with non-SCID TCL or BCL, particularly in regions affected by physician displacement or service fragmentation due to the ongoing war, had inconsistent follow-up. We plan to develop national clinical guidelines for the follow-up and management of children with non-SCID TCL and BCL. Feedback forms for immunologists responsible for screening have been introduced to ensure continuous case monitoring, data collection, and iterative improvement of clinical recommendations.

The other challenge is the lack of confirmatory testing for microdeletion syndromes (e.g., 22q11.2DS), one of the most common syndromic TCLs worldwide, due to limited access to FISH or MLPA testing in some regions.

Overall, our findings validate the feasibility and effectiveness of nationwide NBS for SCID and severe lymphopenias in Ukraine. They highlight the importance of early detection, prompt referral, and timely treatment, especially through HSCT, in improving survival outcomes. Furthermore, population-specific genetic epidemiology (such as *NBN* founder mutations) should be considered when interpreting screening results and planning diagnostic pathways.

### Strengths and limitations of the study

This is the first comprehensive study on nationwide NBS for SCID and other severe lymphopenias in Ukraine. The screening demonstrated high diagnostic sensitivity, low false-positive rates, and successful integration into existing healthcare infrastructure despite a challenging national context.

The program identified previously unrecognized IEIs, such as *IGLL1*-associated B-cell lymphopenias, contributing to the enrichment of national genetic knowledge and patient registries.

Timely HSCT and the high rate of survival in patients with SCID show the clinical impact of early detection.

Limitations of this study include incomplete genetic confirmation in some TCL and BCL cases due to war-related constraints, such as the relocation of patients abroad (resulting in loss to follow-up), limited access to advanced diagnostics in frontline regions, and the displacement of physicians, which makes long-term observation and data collection inconsistent. The other limitation is the underdiagnosis of certain syndromic TCLs, such as 22q11.2DS, likely due to the unavailability of confirmatory genetic testing tools (FISH/MLPA) in some centers or the death of infants before screening results became available.

## Conclusion

The implementation of the nationwide NBS program for SCID and other severe T- and B-cell lymphopenias in Ukraine demonstrated high sensitivity and specificity in detecting SCID, with a low referral rate and high survival rates among diagnosed patients.

The incidence of SCID/leaky SCID in Ukraine is estimated to be approximately 1 in 49,800, which is comparable to data from other countries. Nijmegen breakage syndrome was the most common syndromic cause of non-SCID TCLs. Additionally, the use of KREC assay expanded the diagnostic capacity to include previously unrecognized B-cell disorders, such as *IGLL1*-associated B-cell lymphopenia.

Future efforts should focus on improving genetic confirmation pathways, long-term follow-up of non-SCID cases, and expanding diagnostic capabilities to ensure early detection of syndromic TCL.

## Data Availability

The datasets presented in this study can be found in online repositories. The names of the repository/repositories and accession number(s) can be found in the article/**Supplementary Material**.
