# Supplementary material for: Newborn screening for SCID and severe T- and B-cell lymphopenia in Ukraine: the first analysis of the results, 2022–2025
Source: Front Immunol. 2025 Dec 11;16:1709657. doi: 10.3389/fimmu.2025.1709657 (PMC12738327; doi:10.3389/fimmu.2025.1709657)
Supplement: Supplementary file 1 [file Table1.docx]

**Table S1.** TREC/KREC counts in DBS and immunophenotyping results in newborns with positive NBS

| N | Year of birth | GA | Gender | TREC/KREC counts in the first DBS | | TREC/KREC counts in the second DBS | | CD3 | CD4 | CD19 | NK | |
| --- | --- | --- | --- | --- | --- | --- | --- | --- | --- | --- | --- | --- |
|  |  |  |  | CT | absolute number per PCR reaction | CT | absolute number per PCR reaction | cells/µL | | | | |
|  | **TREC±KREC positive (SCID, leaky SCID)** | | | | | | | | | | | |
| 1 | 2023 | 39 | M | 34.6/30.8 | 818/5,845 | 33.7/31.5 | 384/1,426 | 10 | 13 | 1,208 | 762 | |
| 2 | 2024 | 40 | M | 31.9/34.7 | 310/3,127 | 31.7/34.6 | 355/3,456 | 0 | 0 | 930 | 90 | |
| 3 | 2024 | 40 | M | 33.3/35.4 | 62/23 | 33.9/39.7 | 398/17 | 500 | 360 | 80 | 60 | |
| 4 | 2025 | 40 | F | 30.1/31.3 | 1,649/77 | 31.2/33.7 | 1,874/2,908 | 0 | 0 | 1,100 | 80 | |
| 5 | 2024 | 40 | M | none/none | 0/322 | none/31.7 | 0/145 | 18 | 3 | 223 | 41 | |
| 6 | 2024 | 40 | M | 39.5/36.6 | 0/0 | 44/44 | 6/0 | 120 | 72 | 60 | 1,460 | |
| 7 | 2025 | 37 | M | 35.4/30.1 | 2/89 | none/37.2 | 0/234 | 60 | 10 | 310 | 10 | |
|  | **TREC±KREC positive (non-SCID TCL and other causes)** | | | | | | | | | | | |
| 8 | 2024 | 40 | F | none/30.7 | 0/163 |  |  | 190 | 170 | 820 | | 920 |
| 9 | 2023 | 37 | F | 38.2/37 | 3/3 | 37/36.5 | 4/3 | 710 | 330 | 20 | | 170 |
| 10 | 2023 | 40 | M | 37.2/37.2 | 70/40 | 33.8/37.6 | 42/8 | 670 | 470 | 130 | | 360 |
| 11 | 2024 | 36 | F | 35.1/37.8 | 52/7 | 37/36.8 | 204/323 | 582 | 445 | 51 | |  |
| 12 | 2023 | 39 | M | 32.5/30.4 | 662/8,197 | 36.7/38.3 | 44/11 | 453 | 267 | 163 | | 531 |
| 13 | 2023 | 25 | M | 35.9/37.8 | 1/113 | 38.7/38.2 | 59/117 | 1,100 | 260 | 25 | | 397 |
| 14 | 2024 | 29 | M | 32.4/31.1 | 0/0 | 35.7/30.9 | 430/6,800 | 60 | 40 | 550 | | 100 |
| 15 | 2025 | 29 | M | none/none | 0/0 |  |  | ND | ND | ND | | ND |
| 16 | 2024 | 24 | F | 35/35.4 | 38/158 | 34.5/36.1 | 37/21 | ND | ND | ND | | ND |
| 17 | 2023 | 35 | F | 34.8/38.8 | 90/6 | 33.8/none | 46/0 | 1,534 | 986 | 395 | |  |
| 18 | 2025 | 28 | F | 33.9/34.6 | 142/103 | 33.3/39.2 | 178/6 | ND | ND | ND | | ND |
| 19 | 2023 | 31 | M | 34.3/none | 33/0 | 32.8/37.1 | 36/2 | ND | ND | ND | | ND |
| 20 | 2024 | 35 | M | 34.9/30.7 | 10/281 | 35.7/31.4 | 1/286 | ND | ND | ND | | ND |
| 21 | 2023 | 40 | M | 32.5/37.6 | 123/1 | 30.8/none | 34/0 | 1,907 | 1,549 | 417 | | 566 |
| 22 | 2024 | 34 | M | 35.4/none | 1/0 | 35,8/none | 1/0 | NA | NA | NA | | NA |
| 23 | 2025 | 27 | M | 34.7/34.1 | 36/15 | 38.2/37.4 | 1/2 | 1,280 | 650 | 76 | | 422 |
| 24 | 2024 | 27 | F | 35/37 | 64/12 | 33.8/none | 34/0 | 2,320 | 1,070 | 56 | | 249 |
| 25 | 2024 | 41 | M | 36.6/31 | 15/66 | 35/32.9 | 34/27 | 5,440 | 3,100 | 1,601 | | 3,100 |
| 26 | 2024 | 39 | M | 35.7/35 | 19/13 | 33.4/35 | 98/8 | ND | ND | ND | | ND |
| 27 | 2024 | 26 | M | 37.5/34.5 | 2/17 | 33.7/33.9 | 6/16 | 1,650 | 1,080 | 540 | | 450 |
| 28 | 2025 | 35 | M | 35.8/33.8 | 3/16 | 35.8/31.7 | 10/54 | 1,920 | 1,100 | 1,823 | | 1,041 |
| 29 | 2025 | 29 | M | 35.4/33.5 | 9/14 | 34.8/37.6 | 16/1 | 6,890 | 3,190 | 2,903 | | 1,401 |
| 30 | 2023 | 31 | F | 36.3/38.8 | 6/1 |  |  | 1,270 | 690 | 40 | | 350 |
| 31 | 2023 | 31 | F | 35.8/none | 8/0 |  |  | 1,760 | 840 | 70 | | 550 |
| 32 | 2024 | 26 | M | 36.9/37.2 | 74/15 | 36.8/none | **3/0** | 1,390 | 220 | 1,170 | | 770 |
|  | **KREC positive, TREC normal** | | | | | | | | | | | |
| 33 | 2023 | 41 | M | 31,6/none | 2,518/0 | 30,5/none | 149/0 | 5,256 | 4,236 | 12 | | 450 |
| 34 | 2024 | 40 | M | 30.5/none | 629/0 | /39 | /0 | 4,900 | 2,990 | 0 | | 330 |
| 35 | 2023 | 36 | M | 32.6/none | 3,000/0 | 33.2/39.2 | 7,100/0 | 4,798 | 3,065 | 97 | | 655 |
| 36 | 2023 | 41 | F | 33.3/none | 1,298/0 | 28.4/none | 17,000/0 | 3,360 | 2,570 | 70 | | 440 |
| 37 | 2024 | 37 | F | 30.2/39.1 | 3,543/31 | 27.8/none | 7,000/0 | 4,065 | 2,777 | 83 | |  |
| 38 | 2024 | 41 | M | 30.4/37.3 | 450/2 | 31/none | 178/0 | 5,670 | 3,840 | 30 | |  |
| 39 | 2024 | 40 | M | 28.8/none | 1,700/0 | 32.5/none | 23,700/0 | 4,860 | 3,460 | 30 | |  |
| 40 | 2024 | 36 | M | 33.7/37 | 48/3 | /37.6 | /0 | 3,350 | 2,450 | 10 | | 3 |
| 41 | 2023 | 35 | F | 28/none | 74,370/0 | 28.9/none | 5,100/0 | 3,070 | 2,100 | 40 | | 350 |
| 42 | 2023 | 40 | F | 30.2/36.8 | 10,432/413 | 29.2/37.8 | 17,000/38 | 3,915 | 2,511 | 311 | |  |
| 43 | 2023 | 38 | M | 32.1/none | 25,000/0 | 28/none | 48,000/0 | 3,840 | 2,704 | 964 | | 810 |
| 44 | 2023 | 39 | F | 29.4/35.8 | 28,816/27 | 26.7/35.5 | 124,783/220 | 3,230 | 2,410 | 100 | | 140 |
| 45 | 2023 | 34 | F | 30.7/none | 7,529/0 | 30.5/39.9 | 19,300/80 | 3,371 | 2,629 | 668 | |  |
| 46 | 2024 | 39 | F | 29.3/39.7 | 7,526/13 | 28.8/36.5 | 1,231/2 | ND | ND | ND | | ND |
| 47 | 2025 | 37 | M | 31.1/39.1 | 190/3 | 27.6/35.2 | 6,458/30 | 5,200 | 3,600 | 1,000 | | 340 |
| 48 | 2023 | 39 | F | 28.3/35.5 | 12,000/34 | 29.1/35.6 | 6,988/150 | 5,350 | 4,170 | 1,684 | | 1,007 |
| 49 | 2024 | 38 | M | 29.5/38.6 | 386/0 | 30/37.2 | 533/4 | 5,726 | 3,808 | 60/105 | | 455 |
| 50 | 2024 | 37 | M | 29.5/38.3 | 946/2 | 28.7/38.5 | 2,895/6 | 3,630 |  | 0 | | 514 |
| 51 | 2025 | 40 | M | 29.6/36.1 | 317/208 | 29.6/37.3 | 440/ 1 | 3,200 | 2,430 | 347 | | 1,143 |
| 52 | 2023 | 40 | M | 30.8/38.4 | 73/ 2 | 31.8/37.3 | 75/3 | 3,500 | 2,730 | 210 | | 208 |
| 53 | 2024 | 34 | F | 32.6/36.6 | 38/3 | 32/none | 33/0 | 1,290 | 890 | 662 | | 894 |
| 54 | 2024 | 27 | M | 34.4/none | 66/0 | 32.6/37.7 | 81/1 | 2,890 | 1,880 | 18 | | 1,822 |
| 55 | 2024 | 40 | M | 30.1/none | 520/0 | 29.5/37.7 | 3/10 | 5,740 | 3,920 | 263 | | 628 |
| 56 | 2025 | 38 | M | 31.7/none | 225/0 | 33/none | 215/0 | 4,410 | 3,640 | 1,290 | | 316 |
| 57 | 2023 | 39 | M | 32.6/none | 68/0 |  |  | 1,580 | 1,220 | 30 | | 59 |

DBS – dried blood spot; NBS – newborn screening; GA – gestational age; CT – cycle threshold; PCR – polymerase chain reaction; SCID – severe combined immunodeficiency; TCL – T-cell lymphopenia; M- male; F – female; ND – not done; NA – not available.
