# Supplementary material for: Newborn screening for SCID and severe T- and B-cell lymphopenia in Ukraine: the first analysis of the results, 2022–2025
Source: Front Immunol. 2025 Dec 11;16:1709657. doi: 10.3389/fimmu.2025.1709657 (PMC12738327; doi:10.3389/fimmu.2025.1709657)
Supplement: Supplementary file 2 [file Table2.docx]

**Table S2.** Diagnosis and outcomes in infants with low TREC±KREC levels

| N | Year of birth | GA, week | Weight, gram | Gender | TREC result | KREC result | CD3/CD4/CD19, cells/µL | Genetic testing /affected gene/ DNA positions | Clinical diagnosis | HSCT | Inter-ventions | Outcome |
| --- | --- | --- | --- | --- | --- | --- | --- | --- | --- | --- | --- | --- |
| 1 | 2024 | 40 | 3630 | F | Positive | Negative | 190/170/820 | ***FOXN1***  c.1233delG  heterozygous | TCL, infantile | No | Follow-up | Alive |
| 2 | 2023 | 37 | 2210 | F | Positive | Positive | 710/330/20 | ***NBN***  657del5 | Nijmegen breakage syndrome | No | IgRT | Alive |
| 3 | 2023 | 40 | 2800 | M | Positive | Positive | 670/470/130 | ***NBN***  657del5 | Nijmegen breakage syndrome | No | IgRT | Alive |
| 4 | 2024 | 36 | 1470 | F | Positive | Positive | 582/445/51 | ***NBN***  657del5 | Nijmegen breakage syndrome | No | IgRT | Alive |
| 5 | 2023 | 39 | 3600 | M | Positive | Positive | 453/267/163 | Not done | TCL idiopathic, Sepsis. CID? | No |  | Deceased at 1 mo 7 days |
| 6 | 2023 | 25 | 750 | M | Positive | Positive | 1100/260/25 | Yes /  Not dеtected | CID? Sepsis. Thymic aplasia. Prematurity | No |  | Deceased at 3,5 mos |
| 7 | 2024 | 29 | 2340 | M | Positive | Negative | 60/40/550 | Yes /  Nоt detected | Prematurity, hydrops, CHD, athymia | No |  | Deceased at 1 mo |
| 8 | 2025 | 29 | 1400 | M | Positive | Positive | Not done | *DOCK8* c.5628 G>T, VUS | Di George?  CHD, sepsis, athymia.  Prematurity | No |  | Deceased at 1 mo |
| 9 | 2024 | 24 | 570 | F | Positive | Positive | Not done | Not done | Sepsis.  Prematurity | No |  | Deceased at 1 mo |
| 10 | 2025 | 28 | 1450 | F | Positive | Positive | Not done | Yes/  Not detected | Prematurity.  Neurological disability | No |  | Deceased at 3 mos |
| 11 | 2024 | 34 | 2800 | M | Positive | Positive | Not available | Not done | Prematurity | No |  | Deceased at 7 months |
| 12 | 2024 | 39 | 2200 | M | Positive | Positive | Not done | Yes/  Not detected | CHD | No |  | Deceased at 3 mos |
| 13 | 2023 | 35 | 1480 | F | Positive | Positive | 1534/986/395 | ADA  c.956_960del,  heterozygous | Prematurity , cleft palate | No |  | Alive, well |
| 14 | 2023 | 31 | 1670 | M | Positive | Positive | Not done | Not done | Prematurity | No |  | Lost to follow-up |
| 15 | 2024 | 35 | 2770 | M | Positive | Negative | Not done | Not done | Unknown (refusal) | No |  | Alive, well |
| 16 | 2023 | 40 | 3900 | M | Positive | Positive | 1907/1549/417 | Not done | Idiopathic TCL | No |  | Alive, well |
| 17 | 2025 | 27 | 540 | M | Positive | Positive | 1280/650/76 | *CHD7*  c.5878T>C, VUS | Congenital malformation.  Prematurity | No |  | Alive, severe condition |
| 18 | 2024 | 27 | 600 | F | Positive | Positive | 2320/1070/56 | *FANCI*  c.1622del,  heterozyg | Prematurity | No | IgRT | Alive, well |
| 19 | 2024 | 41 | 3750 | M | Positive | Negative | 5440/3100/1601 | Not done | False positive | No |  | Alive, well |
| 20 | 2024 | 26 | 1000 | M | Positive | Positive | 1650/1080/540 | Yes/  Not detected | Prematurity  BPD, Encepalopathy | No |  | Alive |
| 21 | 2025 | 35 | 1830 | M | Positive | Negative | 1920/1100/1823 | Not done | Prematurity | No |  | Alive, well |
| 22 | 2025 | 29 | 1100 | M | Positive | Positive | 6890/3190/2903 | Not done | Prematurity | No |  | Alive, well |
| 23 | 2023 | 31 | 1360 | F | Positive | Positive | 1270/690/40 | Not done | Prematurity | No |  | Lost to follow-up |
| 24 | 2023 | 31 | 1360 | F | Positive | Positive | 1760/840/70 | Not done | Prematurity | No |  | Lost to follow-up |
| 25 | 2024 | 26 | 1410 | M | Positive | Positive | 1390/220/1170 | Not done | Prematurity | No |  | Alive, well |

GA – gestational age; CID –combined immunodeficiency; TCL – T-cell lymphopenia; M- male; F – female; CHD – congenital heart disease, BPD – bronchopulmonary dysplasia; IgRT – immunoglobulin replacement therapy
