# Supplementary material for: Newborn screening for SCID and severe T- and B-cell lymphopenia in Ukraine: the first analysis of the results, 2022–2025
Source: Front Immunol. 2025 Dec 11;16:1709657. doi: 10.3389/fimmu.2025.1709657 (PMC12738327; doi:10.3389/fimmu.2025.1709657)
Supplement: Supplementary file 3 [file Table3.docx]

**Table S3.** Diagnosis and outcomes in infants with low KREC, normal TREC levels

| N | Year of birth | GA, week | Weight, gram | Gender | CD19, cells/µL | Genetic variants found | Variant | Clinical diagnosis | HSCT | Treatment | Outcome |
| --- | --- | --- | --- | --- | --- | --- | --- | --- | --- | --- | --- |
| 1 | 2023 | 41 | 3240 | M | 12 | ***BTK*** | c.863G>C (VUS) | XLA | No | IgRT | Alive, well |
| 2 | 2024 | 40 | 3500 | M | 0 | ***BTK*** | Del 13-19 exons | XLA | No | IgRT | Alive, well |
| 3 | 20 24 | 27 | 1200 | M | 18 | ***BTK*** | c.1176С>G | XLA | No | IgRT | Alive, well |
| 4 | 2023 | 36 | 2285 | M | 97 | ***IGLL1*** | с.425С>T/ c.258del | BCL | No | IgRT | Alive, well |
| 5 | 2023 | 41 | 4500 | F | 70 | ***IGLL1*** | с.425С>T/c.377T>C | BCL | No | IgRT | Alive, well |
| 6 | 2024 | 37 | 2410 | F | 83 | ***IGLL1*** | c.425C>T/ c.64C | BCL | No | No | Alive, well |
| 7 | 2024 | 41 | 3950 | M | 30 | ***IGLL1*** | c.425C>T/ c.425C>T | BCL | No | IgRT | Alive, well |
| 8 | 2024 | 40 | 3000 | M | 30 | Pending |  | Idiopathic BCL | No | IgRT | Alive, well |
| 9 | 2024 | 36 | 2800 | M | 10/N | *CEBPE* | c.782G>A | Idiopathic transient BCL | No | Unknown | Alive, well |
| 10 | 2023 | 35 | 1700 | F | 40 | Yes/Not detected |  | Idiopathic BCL | No | Unknown | Unknown (moved) |
| 11 | 2023 | 40 | 2900 | F | 311 | Not done |  | Idiopathic transient BCL | No | No | Alive, well |
| 12 | 2023 | 38 | 2500 | M | 810 | Not done |  | False positive | No | No | Alive, well |
| 13 | 2023 | 39 | 3500 | F | 100/N | Not done |  | Idiopathic transient BCL | No | No | Alive, well |
| 14 | 2023 | 34 | 1900 | F | 668 | Not done |  | Prematurity, low weight | No | No | Alive, well |
| 15 | 2024 | 39 | 3320 | F | Not done | Not done |  | Unknown (Refusal) | No | No | Alive, well |
| 16 | 2025 | 37 | 3090 | M | 1000 | Not done |  | False positive | No | No | Alive, well |
| 17 | 2023 | 39 | 3030 | F | 1684 | Not done |  | False positive | No | No | Alive, well |
| 18 | 2024 | 38 | 2750 | M | 60/N | Not done |  | Idiopathic transient BCL | No | No | Alive, well |
| 19 | 2024 | 37 | 3100 | M | 0/N | Yes/Not detected |  | Transient BCL after rituximab | No | IgRT up to 6 mos | Alive, well |
| 20 | 2025 | 40 | 3200 | M | 347 | Not done |  | False positive | No | No | Alive, well |
| 21 | 2023 | 40 | 3150 | M | 210/1720 | Not done |  | Perinatal infection | No | No | Alive, well |
| 22 | 2024 | 34 | 2720 | F | 662 | Not done |  | Prematurity | No | No | Alive, well |
| 23 | 2024 | 40 | 3580 | M | 263 | *PARN* | c.1514 G>A (VUS) | Transient hypohammaglobulinemia | No | IgRT | Alive, well |
| 24 | 2025 | 38 | 2950 | M | 1290 | Not done |  | Perinatal infection | No | No | Alive, well |
| 25 | 2023 | 39 | 3120 | M | 30/N | *CHEK2* | c.470 T>C (P) | Idiopathic transient BCL | No | No | Alive, well |

GA – gestational age; M- male; F – female; XLA – X-linked agammaglobulinemia; BCL – B-cell lymphopenia; IgRT – immunoglobulin replacement therapy; VUS – variant of uncertain significance; P – pathogenic.
